# Obstetric outcomes for women with female genital mutilation at an Australian hospital, 2006–2012: a descriptive study

**DOI:** 10.1186/s12884-016-1123-5

**Published:** 2016-10-28

**Authors:** Nesrin Varol, Angela Dawson, Sabera Turkmani, John J. Hall, Susie Nanayakkara, Greg Jenkins, Caroline S. E. Homer, Kevin McGeechan

**Affiliations:** 1 Discipline of Obstetrics and Gynaecology, Sydney Medical School, University of Sydney, Sydney, NSW Australia; 2 Centre for Midwifery, Child and Family Health, Faculty of Health, University of Technology Sydney, Sydney, NSW Australia; 3 Centre for Clinical Epidemiology & Biostatistics, School of Medicine and Public Health, Faculty of Health and Medicine, University of Newcastle, Sydney, NSW Australia; 4 Department of Obstetrics and Gynaecology, Auburn Hospital, Sydney, NSW Australia; 5 Discipline of Obstetrics and Gynaecology, School of Medicine, Notre Dame University, Sydney, NSW Australia; 6 School of Public Health, University of Sydney, Sydney, NSW Australia

**Keywords:** Female genital mutilation, Obstetric complications, Australia, Data collection

## Abstract

**Background:**

Women, who have been subjected to female genital mutilation (FGM), can suffer serious and irreversible physical, psychological and psychosexual complications. They have more adverse obstetric outcomes as compared to women without FGM. Exploratory studies suggest radical change to abandonment of FGM by communities after migration to countries where FGM is not prevalent. Women who had been subjected to FGM as a child in their countries of origin, require specialised healthcare to reduce complications and further suffering. Our study compared obstetric outcomes in women with FGM to women without FGM who gave birth in a metropolitan Australian hospital with expertise in holistic FGM management.

**Methods:**

The obstetric outcomes of one hundred and ninety-six women with FGM who gave birth between 2006 and 2012 at a metropolitan Australian hospital were analysed. Comparison was made with 8852 women without FGM who gave birth during the same time period. Data were extracted from a database specifically designed for women with FGM and managed by midwives specialised in care of these women, and a routine obstetric database, ObstetriX. The accuracy of data collection on FGM was determined by comparing these two databases. All women with FGM type 3 were deinfibulated antenatally or during labour. The outcome measures were (1) maternal: accuracy and grade of FGM classification, caesarean section, instrumental birth, episiotomy, genital tract trauma, postpartum blood loss of more than 500 ml; and (2) neonatal: low birth weight, admission to a special care nursery, stillbirth.

**Results:**

The prevalence of FGM in women who gave birth at the metropolitan hospital was 2 to 3 %. Women with FGM had similar obstetric outcomes to women without FGM, except for statistically significant higher risk of first and second degree perineal tears, and caesarean section. However, none of the caesarean sections were performed for FGM indications. The ObstetriX database was only 35 % accurate in recording the correct FGM type.

**Conclusion:**

Women with FGM had similar obstetric outcomes to women without FGM in an Australian metropolitan hospital with expertise in FGM management. Specialised FGM services with clinical practice guideline and education of healthcare professionals may increase the detection rate of FGM and improve obstetric management of women with FGM.

**Electronic supplementary material:**

The online version of this article (doi:10.1186/s12884-016-1123-5) contains supplementary material, which is available to authorized users.

## Background

In 2013, the Australian Government’s national strategy recognised the importance of ensuring quality health services for girls and women with FGM and supporting international collaboration to protect girls from this harmful practice and help communities abandon it. Hospitals in Australia are reporting increased presentations of affected women in labour [1]. In Australia, little is known of the prevalence of FGM, or the burden of disease attributed to the practice. This contrasts with available information in other countries such as the United Kingdom (UK), France and Germany where there has been migration from countries where FGM is prevalent. For example, in 2007, 66,000 women were estimated to be living with FGM in the UK [2].

FGM involves partial or total removal of the external female genitalia or other injury to the female genital organs for nonmedical reasons (Table 1) [3].

**Table 1:** WHO Classification of FGM

Type I	Partial or total removal of clitoris and/or prepuce
Type II	Partial of total removal of clitoris and labia minora, with or without excision of labia majora
Type III	Infibulation. Excision of part or all of external genitalia and stitching of the two cut sides together to varying degrees
Type IV	All other harmful procedures to female genitalia for non-medical purposes, for example pricking, piercing, incision, stretching, scraping and cauterisation

More than 200 million girls and women have undergone FGM, with three million at risk each year [4]. Girls may die at the time of the cutting from haemorrhage or septic shock [5], or experience considerable physical, psychological and sexual complications [3, 6–8]. A prospective study by the World Health Organization (WHO) in six African countries has shown that obstetric complications are significantly higher in women with FGM [9]. Women with the more severe form of FGM, i.e., type III or infibulation, had a 30 % higher risk of caesarean section and a 70 % increase in postpartum haemorrhage compared with those without FGM. The perinatal mortality rate was 15, 32 and 55 % higher in women with FGM type I, II and III, respectively [9].

In 2011, about 36,000 women and girls migrated to or were asylum seekers in Australia from countries where FGM is prevalent [10]. The number of refugee intake in Australia, however, is small compared to most of the other high income countries in the world. Moreover, eighty-seven percent of refugees globally are hosted in developing countries [11]. Even though the prevalence of FGM is on the decline globally, and even if this progress in reducing the total number of girls in the countries of prevalence is maintained, the number of girls subjected to FGM will still grow due to population growth [12]. With ongoing wars and conflicts, Australia and other countries will continue to receive migrants, refugees and asylum seekers, some of whom are from countries where FGM is prevalent. A review on current knowledge on cultural change after migration in FGM practice suggests a significant shift towards abandonment [13]. There have been fewer than 50 criminal court cases on FGM in Western countries to date. Whilst it is possible that girls are being cut secretly and without detection, there is far greater evidence that migration is a positive catalyst for attitude change [13].

Currently there are only three hospitals in Australia that have expertise and policies in regards to the care of women with FGM [14–16]. A coordinated national and international approach to inform evidence-based care to provide girls and women affected by this practice better and more cost effective healthcare is required. Accurate data on the prevalence and morbidity of FGM and training of healthcare professionals are prerequisites for this endeavour. Our study examines the impact of FGM on obstetric outcomes for women with FGM who were cared for in a metropolitan Australian hospital with expertise in FGM management. We also assessed the accuracy of data collection on FGM in this hospital.

## Methods

The metropolitan hospital, where our study was based, is a multicultural centre of excellence for the provision of maternity and newborn care services for women with low risk births. There are approximately 1500 births per year at the hospital. The hospital’s patients speak 17 languages, with 67 % of the patients having a non-English speaking background.

The hospital’s specialised FGM clinic follows guidelines by the Royal Australian and New Zealand College of Obstetricians and Gynaecologists (RANZCOG) for optimal maternity care of women with FGM [17]. The presence and type of FGM is documented at the first (booking) visit and a clear care pathway for antenatal care, labour and birth is established and documented for each woman. Counselling, psychological and translation services are provided for women and their families. FGM and its health risks, as well as the legal framework around FGM are discussed. An opportunity is created for women and their partners to explore their perceptions and beliefs regarding FGM. The benefits of deinfibulation are explained and the procedure is offered preferably in the second trimester to allow for vaginal examinations to assess the progress in labour. It is, however, also performed during labour.

Deinfibulation is performed under local anaesthetic. The woman’s vagina is incised open and the cut edges sewn over in a subcuticular fashion for haemostasis and healing. As part of their FGM prevention program, this hospital conducts training on FGM for obstetricians and midwives, general practitioners and nurses specialising in reproductive health. This involves teaching on the sociocultural underpinnings of FGM, cultural competence, identification of the types of FGM, antenatal, intrapartum, and postnatal care with surgical skills of deinfibulation procedure, recognition of other physical and psychosexual complications and appropriate referrals for treatment, and the legal framework around FGM to protect newborn girls. It also provides education sessions for migrant women and their partners on FGM throughout Australia. Antenatal care and definfibulation procedures are provided by the specialist team of midwives and obstetricians.

Our study population were all adult women with singleton pregnancies who gave birth to their babies over 24 weeks at this hospital between 2006 and 2012. All data was accessed retrospectively. The study was approved by the Scientific Advisory Committee and the Human Research Ethics Committee of the Western Local Health District. The data was de-identified for persons prior to analysis. Two sets of data were available containing information about women with FGM during this time. The first dataset is ObstetriX [18], which is completed by midwives at their first visit and after the baby is born. ObstetriX was introduced in NSW in 2005 to replace various public hospital databases to track and manage detailed maternal and neonatal information. This database provides a space to record the presence and type of FGM. The second database has been maintained since 2000 by the Clinical Midwifery Consultant and midwives of the maternity ward who specialise in the management of FGM (the Midwives Database). It is hence considered to have accurate information on FGM status due to the expertise of these midwives. In addition to ObstetriX, the Midwives Database records the deinfibulation procedures the midwives perform for women with FGM type III prior to pregnancy, during their second trimester or in labour.

From each database, we extracted information on whether FGM was recorded, and the type of FGM, for woman who had given birth. From the ObstetriX Database, we extracted the following perinatal outcomes: caesarean section, instrumental birth, medio-lateral episiotomy, genital tract trauma (first and second, third and fourth degree perineal tears), postpartum blood loss of more than 500 ml, admission of the newborn to the special care nursery, and stillbirth. Stillbirth is defined as the complete expulsion or extraction from its mother of a product of conception of at least 20 weeks gestation or 400 g birth weight who did not, at any time after birth, breathe, or show any evidence of life, such as a heartbeat.

In the analysis of prevalence and the perinatal outcomes, if there was a disagreement between the two databases in the recording of FGM, the Midwives Database was deemed to be the correct record due to the FGM expertise of the Clinical Midwife Consultant and midwives. If the type of FGM for a woman differed at different visits, then the most severe type was chosen as the correct type and applied to all obstetric records for that woman.

### Statistical analysis

We cross-tabulated the FGM type recorded in the ObstetriX Database with the FGM type recorded in the Midwives Database for all women’s records between July 2006 and December 2012. We calculated the proportions where FGM was recorded in both datasets and where the FGM type agreed. We calculated the proportion of women with FGM among women who gave birth in the years 2006 to 2012. Among women with and without FGM, we summarised categorical variables using proportions. We fitted Poisson regression models, with general estimating equations to account for subsequent births for women over the years, to estimate the rate of obstetric complications among women with FGM compared with women without FGM. Additionally, for nulliparous women, we fitted a Poisson regression model to assess the association between FGM type and caesarean section rates after adjusting for the potential confounders year of birth, age group, body mass index group, presence of hypertension and diabetes, and smoking status. This analysis was performed only among nulliparous women to remove any effect of previous obstetric history. We were unable to fit adjusted models for the other obstetric outcomes due to their small numbers among nulliparous women.

## Results

### Comparison of FGM recording in ObstetriX and Midwives Databases

There were 9048 maternity records for the period July 2006 to December 2012 for women with singleton pregnancies. ObstetriX recorded FGM in 139 records, whilst the Midwives Database indicated that 180 records were for women with FGM (Table 2). In this period, ObstetriX documented FGM in 73 % (139/191) of all records where FGM was recorded (ObstetriX or Midwives Databases). This proportion increased from 14 % in 2006 to 90 % in 2012.

**Table 2:** Comparison of all obstetric records for women with FGM in ObstetriX and Midwives Databases, 2006–2012

		ObstetriX Database				
		Grade^a^ I-II	Grade III-IV	Not known	Not recorded	Total
Midwives Database	Type I-II	43	2	35	30	110
	Type III-IV	10	20	15	19	64
	In Midwives Database but FGM type not recorded	0	0	3	3	6
	Does not appear in Midwives Database	5	1	5	8857	8868
	Total	58	23	58	8909	9048

^a^The ObstetriX Database codes FGM as either Not known, Grade 1–2 or Grade 3–4, whereas the Midwives Database codes to the more specific type I, II or III

Among the records for women with FGM documented in either database, the ObstretriX record agreed with the Midwives database in only 35 % of cases and the Kappa statistic was 0.28 (95 % CI: 0.18, 0.38). Approximately 39 % of FGM type III recordings in the Midwives Database were recorded incorrectly as grade I-II or “unknown” in ObstetriX. Among women with FGM recorded in ObstetriX, the proportion of “unknown” classification was high at 42 % (Table 2).

### Prevalence and type of FGM, and demographics of women with FGM

There were 196 births from women with FGM at the study hospital between 2006 and 2012 (Table 3). The prevalence of women with FGM varied in year from 1.8 to 2.8 %. The highest prevalence occurred in 2009. The percentages of women with FGM types I, II and III among the known types were 33, 33, and 26 %, respectively.

**Table 3:** Number of women who have FGM recorded and have a maternity record at the study hospital between 2006 and 2012, by year

		Number of women						
FGM recorded	FGM type^a^	2006^b^	2007	2008	2009	2010	2011	2012
Yes	All types	15	25	33	37	29	35	22
	Type III	6	10	10	13	5	13	7
	Type II-III	0	0	1	0	0	0	0
	Type II	2	7	7	12	11	11	7
	Type I-II	0	1	1	3	1	2	0
	Type I	4	5	12	7	11	8	6
	Unknown	3	2	2	2	1	1	2
Percent of women with FGM recorded		2.3	1.8	2.4	2.8	2.2	2.4	1.5
No		631	1390	1344	1307	1306	1447	1427

^a^FGM status and type was determined from examining both the Midwives and ObstetriX Databases. If there was a disagreement between the two databases, the Midwives Database was deemed to be the correct record. If the type of FGM for an individual woman differed at different visits, then the most severe type was chosen as the correct type and applied to all obstetric records

FGM status was grouped as:

Type I/II = Unknown, Type I, Type I-II, Type II

Type III = Type II/III, Type III

^b^2006 includes only July to December

The majority (67 %) of women with FGM were born in East Africa. Almost a quarter (23 %) were from the western and southern regions of Africa, and the remainder from North Africa, the Middle East and Asia. The majority (89 %) of women with FGM type III were from East Africa. Approximately half of the women with FGM from Sudan and Somalia had FGM type III. The prevalence of FGM was the same among women from East Africa and those from western and southern regions of Africa, i.e., 45 %. The prevalence among women from the other countries was less than 1 %.

The age distribution for women with and without FGM was similar, with most women aged between 20 and 34 years (Table 4). A third of the births among women with FGM were to women who had three or more previous deliveries of babies, compared to 17 % among women without FGM. Women with and without FGM were similar in body mass index and the diagnosis of hypertension. Gestational diabetes mellitus was more common among women with FGM type III. Only 1 % of women with FGM were smokers during pregnancy as compared to 5.6 % of women without FGM.

**Table 4:** Demographics of women with FGM recorded at time of obstetric visit at study hospital between 2006 and 2012

	FGM status^a^			FGM status^a^		
	Type III	Type I/II	No	Type III	Type I/II	No
	n	n	n	%	%	%
Age group						
15–19	0	1	326	0.0	0.8	3.7
20–24	8	33	2243	12.3	25.2	25.3
25–29	21	29	2862	32.3	22.1	32.3
30–34	21	43	2216	32.3	32.8	25.0
35–39	11	22	972	16.9	16.8	11.0
40+	4	3	233			
Region of birth						
East Africa^b^	58	73	157	89.2	55.7	1.8
Western/Southern Africa	6	39	56	9.2	29.8	0.6
North Africa and Middle East	0	7	2057	0.0	5.3	23.2
Asia	1	12	2267	1.5	9.2	25.6
Others	0	0	4315	0.0	0.0	48.7
Parity						
0	13	39	3444	0.0	29.8	38.9
1	9	35	2472	20.0	26.7	27.9
2	15	20	1408	13.8	15.3	15.9
3	14	18	808	23.1	13.7	9.1
4 or more	14	19	720	21.5	14.5	8.1
Number of previous preterm or nonviable pregnancies						
0	44	61	5749	67.7	46.6	64.9
1	8	40	2051	12.3	30.5	23.2
2	9	10	710	13.8	7.6	8.0
3	3	14	206	4.6	10.7	2.3
4 or more	1	6	136	1.5	4.6	1.5
BMI						
< 18.5	2	3	370	3.7	2.7	4.8
18.5–24.9	19	44	3982	35.2	40.0	51.2
25.0–29.9	21	43	2037	38.9	39.1	26.2
30+	12	20	1387	22.2	18.2	17.8
Missing data	11	21	1076			
Hypertension						
Essential/Renal	0	0	30	0	0.0	0.3
Gestational/Pre-eclampsia/Eclampsia	3	5	318	4.7	3.8	3.6
No	61	125	8437	95.3	96.2	96.0
Missing data	1	1	67			
Diabetes						
Pre-existing	0	1	46	0	0.8	0.5
Gestational	11	10	655	17.2	7.7	7.5
No	53	119	8078	82.8	91.5	92.0
Missing data	1	1	73			
Smoking status in pregnancy						
No	64	128	8321	100.0	98.5	94.4
Yes	0	2	495	0.0	1.5	5.6
Missing data	1	1	36	100.0	98.5	94.4

^a^FGM status and type were determined from examining both the ObstetriX and Midwives Databases. If there was a disagreement between the two databases, the Midwives Database was deemed to be the correct record. If the type of FGM for an individual woman differed at different visits, then the most severe type was chosen as the correct type and applied to all obstetric records

Women may have more than one obstetric record during the period 2006–2012. Seventy-six percent of women had only one obstetric record (i.e. gave birth once). The average number was 1.3 and the maximum 6

FGM status was grouped as:

Type I/II = Unknown, Type I, Type I-II, Type II

Type III = Type II/III, Type III

^b^East Africa is defined as Tanzania, Burundi, Rwanda, Uganda, Sudan, Ethiopia, Eritrea, Djibouti, Somalia, and Kenya. Western and Southern African countries include Sierra Leone, Liberia, Guinea, Nigeria and the Democratic Republic of Congo. North Africa and the Middle East categories include Egypt, Iran, Iraq, Saudi Arabia and Yemen. Women from Asia were from Pakistan, Sri Lanka, Indonesia and Singapore

### Maternal and neonatal outcomes for women with and without FGM

Table 5 displays the rates of maternal and neonatal outcomes for women with different types of FGM and for women without FGM. Almost all women with FGM type III had deinfibulation performed either prior to or during pregnancy. Only 9 % were deinfibulated during labour.

**Table 5:** Perinatal outcomes for women who have FGM recorded and have an obstetric record at study hospital between 2006 and 2012

	FGM status			FGM status			
	Type III	Type I/II	No	Type III	Type I/II	No	
	n	n	n	Rate per 100 births (95 % CI)	Rate per 100 births (95 % CI)	Rate per 100 births (95 % CI)	*P*-value
Outcome							
Caesarean section^a^							
Yes	16	36	1706	26.5 (16.6, 42.4)	32.9 (24.5, 44.0)	20.4 (19.5, 21.4)	0.0041
No	49	95	7145				
Instrumental births^a^							
Yes	1	8	574	1.6 (0.2, 11.2)	6.2 (2.9, 13.1)	6.5 (6.0, 7.1)	0.36
No	64	123	8277				
Episiotomy (excluding anterior episiotomy)							
Yes	7	17	946	10.5 (5.0, 21.8)	12.7 (7.5, 21.5)	10.9 (10.2, 11.5)	0.84
No	58	114	7906				
3^rd^ and 4^th^ degree perineal tear^b^							
Yes	0	0	85	0.0	0.0	1.0	^b^
No	65	131	8767				
Genital tract trauma							
Yes	4	2	99	6.0 (2.4, 14.8)	1.4 (0.4, 5.6)	1.1 (0.9, 1.4)	0.0015
No	61	129	8753				
Postpartum blood loss > 500 ml							
Yes	1	7	615	1.6 (0.2, 11.6)	5.6 (2.7, 11.4)	7.0 (6.5, 7.6)	0.29
No	64	124	8237				
Low birth weight (<2500 g)^a^							
Yes	2	4	259	3.5 (0.9, 13.8)	3.1 (1.2, 8.1)	3.0 (2.6, 3.4)	0.97
No	63	127	8590				
Admission to special care nursery							
Yes	6	16	616	9.0 (3.9, 20.7)	12.0 (7.5, 19.2)	7.0 (6.5, 7.6)	0.07
No	59	115	8236				
Stillbirth^c^							
Yes	1	1	39	1.5 (0.2, 10.9)	0.8 (0.1, 5.3)	0.4 (0.3, 0.6)	0.41
No	64	130	8812				

^a^One woman’s record had missing mode of birth and three records had missing birth weight

^b^Statistical model could not be fitted due to small numbers

^c^One woman’s record had missing stillbirth indicator

Maternal and neonatal outcomes for women with FGM were similar to those without FGM, except for statistically significant higher rates of caesarean section and first and second degree perineal tears. The latter was more common among women with FGM type III as compared to types I and II (Table 5).

### Caesarean section rates amongst nulliparous women with FGM

Nulliparous women with FGM types III and I/II, and those without FGM had a caesarean section rate of 46 % (*n* = 6), 40 % (*n* = 15), and 20 % (*n* = 697), respectively (Table 6).

**Table 6:** Proportion (%) of nulliparous women who had a caesarean section, by FGM type for women who have an obstetric record for the period 2006 to 2012 at study hospital

	FGM Status			FGM Status		
	Type III	Type I/II	No	Type III	Type I/II	No
	n	n	n	%	%	%
Outcome						
Caesarean section^a^						
Yes	6	15	697	46.2	38.5	20.3
No	7	24	2744			

^a^One obstetric record had missing mode of birth

Among nulliparous women, the crude relative risk of having a caesarean section for women with FGM type III compared to women without FGM was 2.3 (95 % CI: 1.3–4.1), and the adjusted relative risk was 1.5 (0.9–2.6). Among nulliparae, the crude relative risk of having a caesarean section for women with FGM Type I/II compared to women without FGM was 1.8 (95 % CI: 1.2–2.9), and the adjusted relative risk was 1.6 (CI 1.0–2.6). Nulliparous women with FGM had higher rates of caesarean section than women without FGM (*P* = 0.037).

Despite the statistically significant higher rates of caesarean section among women with FGM, none of the caesarean sections had FGM as an indication.

### Indications for caesarean sections for women with FGM

Sixty-three percent of all caesarean sections for women with FGM (multiparae and nulliparae) were emergency and the remainder elective. Amongst emergency operations, 78 % had FGM type I/II. The indications were non-reassuring fetal heart rate trace with or without abnormal fetal scalp lactate (65 %), failure to progress (19 %), pre-eclampsia (9 %), placental abruption (3 %) and placenta praevia (3 %). Sixty-three percent underwent a caesarean section at cervical dilatation of up to 4 cm.

The indications for all elective caesarean sections were previous caesarean section (74 %, *n* = 14), malpresentation (21 %, *n* = 4), and meconium-stained liquor with no labour (5 %, *n* = 1).

Amongst nulliparae, 81 % were emergency caesarean sections. Most of them (71 %) were for non-reassuring fetal heart rate trace. Two-thirds of these were inductions of labour for postdates, 75 % of whom underwent caesarean section at cervical dilatation of 2-4 cm. See (Additional file 1: Table S7) for the original data used to perform the above analysis.

## Discussion

### Main findings

The prevalence of women with FGM who gave birth at the metropolitan hospital in Australia between 2006 and 2012, was 2 to 3 %. The accuracy of ObstetriX database in collecting data on the presence of FGM increased from 14 to 90 % over six years. However, it was only 35 % correct in identifying the type of FGM. Ninety percent of women with FGM were born in the countries of Africa. Two thirds of all women with FGM had types II and III. Ninety percent of women with type III were from East Africa. Half of the women from Somalia and Sudan had FGM type III.

All women with FGM III were deinfibulated for the birth of their baby unless an elective caesarean section was performed. Caesarean section and genital tract trauma were more common in women with FGM. The latter was higher amongst women with FGM type III as compared to types I and II. None of the women’s caesarean sections were directly indicated for FGM.

### Strengths and limitations

The main strength of the study is that it is the first available data on the number of women with FGM giving birth at a hospital in Australia. It is also the first information on obstetric and neonatal outcomes in women with FGM in this country. Moreover, our study is one of few in the literature to stratify obstetric complications for FGM type. Our study highlights the importance of the need for hospitals and healthcare professionals with expertise in caring for women with FGM.

The small number of women with FGM in the obstetric outcomes categories limited statistical analysis. Combining datasets from multiple hospitals across Australia and internationally may address this issue.

### Interpretation

In 2004, 1.4 % of babies born in all maternity services in England and Wales were to women affected by FGM [19]. Our figure of 2 to 3 % for the study hospital would be lower nationally, as this hospital is known for its FGM expertise. Women born in countries where FGM is prevalent, live near it, and hence are referred to this hospital. Eighty-nine percent of women who were born in these countries, lived in major cities in Australia in 2011 [20].

In keeping with our findings of ObstetriX recording of FGM type, a study in Switzerland reported that FGM identification were missed in 37 % and the type misclassified in 23 % [21]. There is no data on FGM prevalence in Australia. Similarly, in the European Union, data relevant to FGM is neither systematically collected nor centrally stored [19]. Accurate data is important in developing evidence-based policies to appropriately implement prevention programmes, health services, monitor their effectiveness, and assess how integration processes affect the practice [19]. It is important to again stress the fact that a radical change occurs in families to oppose and/or abandon FGM after migration [13], and we can expect a very low number of children being cut in their new countries. Hence, while it is paramount to prevent and protect girls from FGM in a similar way as we do with child abuse, it is also important to focus on the issues surrounding social integration and education to aid the cultural change.

The WHO study found that obstetric complications increased with severity of FGM type [9]. The study centres varied from isolated rural hospitals to tertiary teaching hospitals in capital cities. In our study, most of the women had the more severe types II and III and obstetric outcomes were similar to women without FGM except for caesarean section and first and second degree perineal tears. The findings of our study are in keeping with those of other high-income countries, which show that high quality obstetric care with expertise in FGM can minimise obstetric complications [22–25].

A study in Switzerland found statistically higher incidences of caesarean section and third degree perineal tears in women with FGM III [24]. The majority of their 122 patients had been deinfibulated. The main reason for the emergency caesarean sections, however, was the inability to perform a vaginal examination in labour in women with FGM III who had not been deinfibulated. We do not know whether the caesarean section rate would still have been higher if all women had been deinfibulated and allowed to continue to labour. There was no difference in the length of labour between women with and without FGM. The latter finding was supported by a study of women who underwent deinfibulation at vaginal delivery at a tertiary referral hospital in Saudi Arabia [26]. There were no differences in obstetric and perinatal outcomes. No caesarean section was performed for the indication of FGM. The strength of that study was that the women were matched to those of the same nationalities. A UK study showed that reversal of FGM III significantly reduced the increased risk of caesarean delivery seen with multiparae who have FGM III [27]. A Swedish study examined 68 nulliparous women who had mainly FGM III and were deinfibulated in labour [23]. It found that women with FGM even had a lower risk of prolonged labour.

On the other hand, in keeping with findings from the WHO study [9], the above obstetric outcomes have been found to be different in low-income countries. A study of 85 women in Burkina Faso again showed that women with FGM had a higher risk of caesarean section [28]. The indications were cephalopelvic disproportion and prolonged labour with fetal distress. The study also found higher risks of episiotomy, neonatal resuscitation and stillbirth in women with FGM. These findings were supported by a prospective study in The Gambia [29]. FGM did not affect the main reason of cephalopelvic disproportion for caesarean section. The authors posited that caesarean sections were performed preventively for severe abnormal scarring and/or synaechiae in women with FGM II [29].

Studies of Somali-born women in high-income countries (HIC) of migration reveal significantly adverse perinatal outcomes as compared to receiving country-born women [17, 19, 20, 30]. A meta-analysis of these women in six countries showed that they were more likely to labour without any analgesia or epidural [17]. Language and communication barriers were considered to be contributing factors to the differences in the use of pain relief [17]. These may then also affect the increased number of caesarean section, which occurred despite the fact that Somali women prefer vaginal birth and have an aversion to caesarean section [19, 26, 27]. This fear of caesarean section understandably arises from having lived in a country with a high maternal mortality rate. One of the reasons could be late presentation of women to hospital when a caesarean section is performed but the complications are too advanced to safe a woman’s life. Moreover, women may die from uterine rupture with subsequent pregnancies following a caesarean section, as there may be no access to appropriate obstetric care. These women’s beliefs regarding safe birth need to be explored and addressed early in their antenatal care. A qualitative study of pregnancy and childbirth experiences in Somalian women in Sweden proposes a relationship between adverse perinatal outcome and cultural factors [31]. A comparison of these studies from HIC with otherwise high-quality obstetric care with those of the HIC above suggests that culturally sensitive care and FGM expertise by the obstetrician and midwife are likely to play important roles in providing optimal perinatal care. The higher caesarean section rate in our study is unclear. A prospective multicentre study in countries of migration comparing women with and without FGM of same ethnicities would be required to provide that information. Moreover, the study hospitals would need to provide similar standards of obstetric care and FGM expertise.

Our study hospital underlines the importance of integrating specialised FGM units into hospitals that care for women with FGM. They would provide holistic healthcare, including gynaecological and obstetric care with deinfibulation procedures, integrated with culturally appropriate counseling, psychological, sexual and social support services. There need to be referral pathways for healthcare professionals, hospitals, schools, social work, child protection, immigration and judicial sectors, the police and nongovernment organisations working with affected communities. Training in FGM and its legal and health implications for women and their families can be integrated in these sectors.

In Australia and other countries where women with FGM live, there is a concern about the cultural competence, experience and training of healthcare professionals in the management of FGM [32–36]. The comparison of the ObstetriX and specialised Midwives Databases in our study show a significant improvement in the accuracy of FGM recording from 14 to 90 % over six years, indicating increasing knowledge of midwives through teaching of the Clinical Midwifery Consultant. Our study highlights the importance of education and training of healthcare providers in FGM diagnosis and care. These may be formally incorporated into undergraduate and postgraduate nursing and medical programmes.

## Conclusion

Women with FGM had similar obstetric outcomes to women without FGM in an Australian metropolitan hospital with FGM expertise. There was an increased rate of caesarean section and genital tract trauma of first and second degree perineal tears. The indications for the caesarean sections were not FGM.

There was significant improvement in midwifery expertise in FGM diagnosis over seven years with formalised training and education at the study hospital. Our findings underline the benefits of providing care for women with FGM in a specialised centre.

## Additional file

Additional file 1: Table S7. Indications for caesarean sections for nulliparae and multiparae with FGM who had caesarean sections. (DOC 38 kb)

## Abbreviations

CS: Caesarean section
FGM: Female genital mutilation
FHR: Fetal heart rate
HIC: High-income countries
RANZCOG: Royal Australian and New Zealand College of Obstetricians and Gynaecologists
UK: United Kingdom
WHO: World Health Organization

## References

[1] Bourke E. Female Circumcision happening in Australia. ABC News. 2010. http://www.abc.net.au/news/stories/2010/02/06/2812147.htm. Accessed 1 Jun 2015.

[2] van Vossole A, Middelburg MJ, Arnaut C, Leye E, Deblonde J, Mergaert L, et al. Female genital mutilation in the European Union and Croatia. Report. 2013. http://eige.europa.eu/rdc/eige-publications/female-genital-mutilation-european-union-report. Accessed 26 Jul 2016.

[3] World Health Organization. Eliminating female genital mutilation: An interagency statement–OHCHR, UNAIDS, UNDP, UNECA, UNESCO, UNFPA, UNHCR, UNICEF, UNIFEM, WHO. 2008. http://www.who.int/reproductivehealth/publications/fgm/9789241596442/en/. Accessed 26 Jul16.

[4] United Nations Children’s Fund. Female Genital Mutilation/Cutting: A global concern, UNICEF, New York. 2016. http://www.unicef.org/media/files/FGMC_2016_brochure_final_UNICEF_SPREAD.pdf. Accessed 11 Feb 2016.

[5] Yoder P, Abderrahim N, Zhuzhuni A (2004). Female genital cutting in the Demogragphic and Health Surveys: a critical and comparative analysis. DHS Comparative reports No 7.

[6] Almroth L, Bedri H, El Elmusharaf S, Satti A, Idris T, Hashim MSK (2005). Urogenital complications among girls with genital mutilation: A hospital based study in Khartoum. Afr J Reprod Health.

[7] Talle A, Hernlund Y, Shell-Duncan B (2007). Female circumcision in Africa and beyond: the anthropology of a difficult issue. Transcultural bodies: female genital cutting in global context.

[8] Elnashar RA, Abdelhady R (2007). The impact of female genital cutting on health of newly married women. Int J Gynaecol Obstet.

[9] Banks E (2006). Female genital mutilation and obstetric outcome: WHO collaborative prospective study in six African countries. Lancet.

[10] Australian Bureau of Statistics: Migration, Australia, 2010–11. Canberra/ABS. Cat. No. 3412.0; 2010. http://www.abs.gov.au/ausstats/abs@.nsf/Products/07C4285C66219C10CA257A5A00120A94?opendocument. Accessed 26 Jul 2016.

[11] UNHCR report shows leap in asylum applications for industrialized countries. UNHCR. The UN Refugee Agency. 2014. http://www.unhcr.org/532afe986.htm. Accessed 30 May 2015.

[12] Female genital mutilation/cutting: What might the future hold? 2014. http://reliefweb.int/sites/reliefweb.int/files/resources/FGM-C_Report_7_15_Final_LR.pdf. Accessed 16 Jun 2015.

[13] Johnsdotter S, Essén B (2016). Cultural change after migration: Circumcision of girls in Western migrant communities. Best Practice & Research. Clin Obstet Gynecol.

[14] Auburn Hospital. Western Sydney Local Health District. NSW Government Health. http://www.wslhd.health.nsw.gov.au/Auburn-Hospital. Accessed 26 Jul 2016.

[15] Female genital mutilation - Women and Newborn Health Service, King Edward Memorial Hospital. Clinical Guidelines - Obstetrics & Midwifery. 2014. http://www.kemh.health.wa.gov.au/development/manuals/O&G_guidelines/sectionb/1/b1.3.pdf. Accessed 26 July 2016.

[16] African’s Women Clinic. The Royal Women’s Hospital. Victoria, Australia. https://www.thewomens.org.au/health-professionals/sexual-reproductive-health/african-womens-clinic/. Accessed 26 Jul 2016.

[17] Small R, Gagnon A, Gissler M, Zeitlin J, Bennis M, Glazier RH (2008). Somali women and their pregnancy outcomes postmigration: data from six receiving countries. Int J Obstet Gynecol.

[18] Meridian Health Informatics. ObstetriX - Maternity Care Solution. http://www.meridianhi.com/index.php/obstetrix. Accessed 26 Jul 2016.

[19] Vangen S, Stoltenberg C, Johansen REB, Sundby J, Stray-Pederson B (2002). Perinatal complications among ethnic Somalis in Norway. Acta Obstet Gynecol Scand.

[20] Johnson E (2005). Increased risk of adverse pregnancy outcome among Somali immigrants in Washington State. Am J Obstet Gynecol.

[21] Berggren V, Ahmed SM, Hernlund Y, Johansson E, Habbani B, Edberg A-K (2006). Being victims or beneficiaries? Perspectives on female genital cutting and reinfibulation in Sudan. Afr J Reprod Health.

[22] Amusan OA, Asekun-Olarinmoye EO (2006). Knowledge, beliefs, and attitudes to female genital mutilation (FGM) in Shao Community of Kwara State, Nigeria. Int Q Community Health Educ.

[23] Essen B, Sjoberg N-O, Gudmundsson S, Ostergren P-O, Lindqvist P (2004). No association between female circumcision and prolonged labour: a case control study of immigrant women giving birth in Sweden. Eur J Obstet Gynecol Reprod Biol.

[24] Wuest S, Raio L, Wyssmueller D, Mueller M, Stadlmayr W, Surbek DV (2009). Effects of female genital mutilation on birth outcomes in Switzerland. BJOG.

[25] Abdelshahid A, Campbell C (2015). “Should I circumcise my daughter?” Exploring diversity and ambivalence in Egyptian parents’ social representations of female circumcision. J Community Appl Soc Psychol.

[26] Brown E, Carroll J, Fogarty C, Holt C (2010). “They get a c-section…They gonna die”: Somali women’s fears of obstetrical interventions in the United States. J Transcult Nurs.

[27] Hernandez P, Liamputtong P (2007). Sensing vulnerability, seeking strength: Somali women and their experiences during pregnancy and birth in Melbourne. Reproduction, Childbearing and Motherhood: A Cross-Cultural Perspective.

[28] Frega A, Puzio G, Maniglio P, Catalano A, Milazzo GN, Lombardi D (2013). Obstetric and neonatal outcomes of women with FGM I and II in San Camillo Hospital, Burkino Faso. Arch Gynecol Obstet.

[29] Al-Khulaidi GA, Nakamura K, Seino K, Kizuki M (2013). Decline of supportive attitudes among husbands toward female genital mutilation and its association to those practices in Yemen. PLoS One.

[30] Herral N, Olevitch L, Dubois D, Terry P, Thorp D, Kind E (2004). Somali refugee women speak out about their needs for care during pregnancy and delivery. J Midwifery Womens Health.

[31] Essén B, Johnsdotter S, Hovelius B, Gudmundsson S, Sjöberg NO, Friedman J (2000). Qualitative study of pregnancy and childbirth experiences in Somalian women resident in Sweden. BJOG.

[32] Murray L, Windsor C, Parker E, Tewfik O (2010). The experiences of African women giving birth in Brisbane, Australia. Health Care Women Int.

[33] Dawson A, Turkmani S, Fray S, Nanayakkara S, Varol N, Homer C (2015). Evidence to inform education, training and supportive work environments for midwives involved in the care of women with female genital mutilation: A review of global experience. Midwifery.

[34] Dawson A, Homer CSE, Turkmani S, Black K, Varol N (2015). A systematic review of doctors’ experiences and needs to support the care of women with female genital mutilation. Int J Gynecol Obstet.

[35] Cappon S, L’Ecluse C, Clays E, Tency I, Leye E (2015). Female genital mutilation: knowledge, attitude and practices of Flemish midwives. Midwifery.

[36] Leye E, Ysebaert I, Deblonde J, Claeys P, Vermeulen G, Jacquemyn Y (2008). Female genital mutilation: knowledge, attitude and practices of Flemish gynaecologists. Eur J Contracept Reprod Health Care.

