# Supplementary material for: Obstetric outcomes for women with female genital mutilation at an Australian hospital, 2006–2012: a descriptive study
Source: BMC Pregnancy Childbirth. 2016 Oct 28;16:328. doi: 10.1186/s12884-016-1123-5 (PMC5084319; doi:10.1186/s12884-016-1123-5)
Supplement: Additional file 1: Table S7. — Indications for caesarean sections for nulliparae and multiparae with FGM who had caesarean sections. (DOC 38 kb) [file 12884_2016_1123_MOESM1_ESM.doc]

Additional File 1

|  |  | Nulliparae with FGM who had CS* (n=21) | | | | Multiparae with FGM who had CS (n=30) | | | |
| --- | --- | --- | --- | --- | --- | --- | --- | --- | --- |
|  |  | Emergency CS (n=17) | | Elective CS (n=4) | | Emergency CS (n=15) | | Elective CS (n=15) | |
|  |  | FGM Type III | FGM Type I/II | FGM Type III | FGM Type I/II | FGM Type III | FGM Type I/II | FGM Type III | FGM Type I/II |
| Primary indication for CS | Non-reassuring FHR** trace | 2 | 10 |  |  | 2 | 7 |  |  |
|  | Pre-eclampsia |  | 1 |  |  | 1 | 1 |  |  |
|  | Failure to progress | 1 | 3 |  |  |  | 2 |  |  |
|  | Malpresentation |  |  | 2 | 1 |  |  | 1 |  |
|  | Meconium stained liquor, no labour |  |  | 1 |  |  |  |  |  |
|  | Placental abruption |  |  |  |  |  | 1 |  |  |
|  | Previous CS |  |  |  |  |  |  | 3 | 11 |
|  | Placenta praevia |  |  |  |  | 1 |  |  |  |
| No. of women who had >1 indication for CS |  |  | 2 *** |  |  | 4 **** |  |  |  |
|  | out of non-reassuring FHR trace (n=12), no. of women with induction of labour for postdates (41+/40) | 2 | 8 |  |  | 0 | 0 |  |  |
|  | cervical dilatation at CS: up to 4cm | 1 | 9 |  |  | 4 | 6 |  |  |

Table 7. Indications for caesarean sections

* caesarean section

** fetal heart rate

*** combination of pre-eclampsia, non-reassuring FHR trace, FTP

**** combination of non-reassuring FHR trace, FTP, previous CS, pre-eclampsia, placenta praevia
